# Predictors of treatment outcome for individuals with alcohol use disorder with a goal of controlled drinking

**DOI:** 10.1186/s13722-024-00443-z

**Published:** 2024-02-22

**Authors:** Stina Ingesson-Hammarberg, Nitya Jayaram-Lindström, Anders Hammarberg

**Affiliations:** Centre for Psychiatry Research, Department of Clinical Neuroscience, Karolinska Institutet, & Stockholm Health Care Services, Region Stockholm, Norra Stationsgatan 69, 113 64 Stockholm, Sweden

**Keywords:** Behavioral self-control training, Motivational enhancement therapy, Controlled drinking, Predictor study, Alcohol use disorder

## Abstract

**Background:**

Research is lacking on predictors of outcome for the treatment of alcohol use disorder (AUD) with a goal of controlled drinking (CD). The aim of the study was to investigate one-year outcomes of an RCT, investigating Behavioral Self-Control Training (BSCT) and Motivational Enhancement Therapy (MET) and predictors of positive outcome for weekly alcohol consumption, CD and symptom reduction in AUD.

**Methods:**

This study is based on secondary analyses from a randomized controlled trial including 250 individuals with AUD (52% men) recruited from three specialized addiction clinics in Stockholm, Sweden. Linear and logistic mixed regression models were used for outcomes at 52 weeks, and linear and logistic regression models for the predictor analyses.

**Results:**

BSCT was superior to MET for the change between baseline to 52 weeks for the outcome of CD, defined as low-risk drinking below ten standard drinks per week for both genders (*p* = 0.048). A total of 57% of individuals in BSCT attained a level of CD, as opposed to 43% in MET. Females were significantly better in attaining low-risk drinking levels compared to men. The predictor for obtaining CD and reducing weekly alcohol consumption, was a lower baseline alcohol consumption. Predictors of symptom reduction in AUD were lower baseline level of AUD, and a lower self-rated impaired control over alcohol consumption.

**Conclusions:**

BSCT was superior to MET in obtaining CD levels, and women were superior to men for the same outcome. The study corroborated baseline consumption levels as an important predictor of outcome in CD treatments. The study contributes with important knowledge on key treatment targets, and knowledge to support and advice patients in planning for treatment with a goal of controlled drinking.

*Trial registration*: The original study was registered retrospectively at isrtcn.com (14539251).

## Introduction

Alcohol use disorder (AUD) is one of the largest preventable contributors to the global burden of disease [1, 2], as well as one of the most under-treated psychiatric disorders, with an estimated treatment coverage of 10–20% globally [3, 4]. One of the major contributors to this treatment gap in AUD, is the lack of available non-abstinence-oriented treatments, coupled with the perceived expectation among people with AUD, of not being able to choose a non-abstinence treatment goal when seeking help within a health care setting [5, 6]. Treatment goals aiming for reducing alcohol consumption rather than abstinence are commonly referred to as non-abstinence, reduced risk drinking, moderation, harm reduction, or controlled drinking (CD). In the current paper, the term CD is used to specify treatments that aim to reduce alcohol consumption to a stable level of drinking within predefined limits of low-risk consumption [7]. Other definitions of CD involve a consumption pattern with no or few heavy drinking episodes, defined as not exceeding a specific number of drinks, commonly three/four (women/men) standard drinks, per drinking occasion [8, 9].

One behavioral interventions for CD is Behavioral Self-Control Training (BSCT) [10]. BSCT is a treatment based on cognitive behavior therapy, and involves components such as goal setting, identification of risk situations, moderation strategies, and relapse prevention [11, 12]. Another treatment method which allows for a treatment goal of CD is Motivational Enhancement Therapy (MET). MET is a treatment for AUD based on motivational interviewing [13–15] which encourages patients to define their own treatment goal, thus applicable to both a goal of CD and abstinence.

Behavioral interventions for the treatment of AUD have shown similar effects across a range of theoretically different approaches, with small effect sizes when treatments are compared to an active comparator [16]. Moreover, behavioral interventions aiming for CD have been suggested to be non-inferior in reducing alcohol consumption compared to abstinence-oriented treatments [7, 10]. Recent research suggests that a large proportion of individuals with AUD prefer a CD treatment goal (82–91%) [17–19]. However, the proportion of individuals who are able to obtain a level of CD vary substantially between studies. In a systematic evaluation, Miller proposed that between 25 and 90% of treatment seeking individuals, including both problem drinkers and individuals with AUD treated with a CD intervention, were able to achieve CD [20]. The range in outcomes may be due to large variation in the definition of CD, and heterogeneity in patients with AUD included in the different studies.

Predictor analyses of treatment outcomes for AUD may contribute to the knowledge on which specific intervention leads to a favorable treatment outcome for a given patient, and to more precisely enable the identification of non-responders to treatment. Such studies may also serve as a source of information for clinicians and patients in the process of goal setting, at the beginning of treatment. A number of studies have examined predictors for a favorable outcome in AUD treatment [21–25]. The most consistently supported predictors in behavioral interventions are clinical characteristics such as high alcohol-related self-efficacy [26, 27], low dependence severity [28–32], low psychiatric comorbidity [33–35], high motivation for change [36, 37], a self-selected treatment goal [38–40]. Further, a lower level of impaired control over alcohol consumption, low situational craving [41–43], and higher treatment attendance have shown to be associated with more favorable outcomes [44]. Noteworthy is though, that several clinically relevant predictors have shown mixed results as predictors for treatment outcomes. Having previous treatment attempts has shown to predict both favorable and unfavorable alcohol related outcomes [21]. With regard to patient demographics, some factors have shown mixed results in predicting treatment outcome. One such example is gender, where female gender in some studies has shown to be a risk factor, while in other studies being female was a protective factor of relapse into drinking [21, 45–48]. Further, being of older age has shown to predict larger reductions in number of drinking days and days with heavy drinking among women in treatment for AUD as compared to younger individuals, but the same pattern has not been corroborated for men [21, 49].

There are only a few well supported predictors for a favorable outcome specifically related to CD treatment. Among the most well studied factors, is dependence severity, in which a lower degree of dependency has shown to be associated with a higher ability to attain a lower consumption level than individuals with a higher symptom burden [25, 29, 32, 50]. Further, a high reported motivation for change, has been identified as a predictor of attaining CD [30, 36, 38–40, 51, 52]. In addition, stating a goal of CD, and having an expectation to attain a CD goal have both been found to be predictive of CD [40, 53–55]. In more recent studies, Witkievitz and colleagues have published several studies based on pooled data from Project MATCH, COMBINE-, and the United Kingdom Alcohol Treatment Trial (UKATT), with the aim of increasing the acceptance of non-abstinence outcomes in research and treatment. These studies have for example shown that a lower level of alcohol consumption prior to and during treatment, being employed, having a supporting network and a low degree of mental health problems are factors associated with low-risk drinking [29, 32].

Despite the important contributions made by prior research investigating predictors for outcome in AUD with CD treatment, there are limitations worth mentioning. Several studies have applied outdated diagnostic instruments, included small and non-randomized samples, and used disparate outcome measures. Further, although well powered, the recent studies by Witkiewitz and colleagues [29, 32, 56–58] were not based on trials that included any formal treatment supporting a CD goal, although it was optional to state a controlled drinking goal in both UKATT and the COMBINE trial. Further, there is large disparity in outcome measures applied in the studies evaluating treatment efficacy and predictors, e.g. reduction in heavy drinking days, reduction in days with drinking, percentage of days abstinent and consumption below specified limits. These shortcomings in previous studies decreases the ability to interpret the results regarding predictors for CD.

Taken together, there is a need for further investigation of the scientific evidence of treatment methods for CD, as well as potential predictors of positive outcome in AUD, with the exclusive focus on CD goals. In the current study, the aim was therefore to investigate the long-term efficacy of BSCT and MET for individuals with AUD with a goal of CD, with the hypothesis that BSCT would be superior to MET in reducing alcohol consumption and obtaining CD. Further, the aim was to identify potential predictors of outcome one-year after initiating treatment. Based on previous studies on predictors of CD, we hypothesized that high motivation for change, less severe AUD, lower levels of alcohol consumption at baseline, a lower level of self-rated impaired control and craving, would predict a favorable outcome regarding reduction in alcohol consumption, CD and AUD severity at 52 weeks. Among demographic factors, we included age, gender, and mean income with no specified direction, as they received some support of being predictors of outcome of AUD treatment in general. Other clinical characteristics included as potential predictors of treatment outcome were previous treatment experiences, and family history of AUD which were hypothesized to be predictive of non-favorable outcomes, as they may be expected to be associated with heavier symptom burden and longer time with the problem. For more exploratory purposes, we added receiving treatment on video, more sessions attended, and difficulties in emotion regulation, factors which to our knowledge have not previously been investigated as predictors of outcome in treatment for AUD.

## Methods

### Design

The current study was a prospective cohort study, nested within a randomized controlled trial (RCT) conducted within the Stockholm Centre for Dependency disorders in Stockholm, Sweden. The aim of the RCT was to investigate if BSCT was superior to MET in reducing alcohol consumption, in a sample of 250 individuals with AUD. Results for the primary outcome (26 weeks) have been reported elsewhere, please see for further information of the trial in [59]. The trial had a between groups parallel design with a 1:1 allocation ratio and was carried out between 2017-08-14 and 2022-12-01. The study involved five measuring points, including baseline and follow-up at 12, 26, 52 and 104 weeks after inclusion. The current paper covered data from baseline and 52 weeks.

### Participants and procedure

Participants were self-referred between 2017-08-15 and 2020-12-01 and were recruited among newly admitted patients at the clinic, and by advertisements in social media. Thereafter, participants were assessed for the following inclusion criteria by a trained research coordinator in a scheduled meeting, according to a standardized screening protocol: age 18–70 years, stable housing in the Stockholm region, fulfilling a diagnosis of AUD, reporting an alcohol consumption on at least 30 of the last 90 days, stating controlled drinking as the desired treatment goal, and willingness to give informed consent. The exclusion criteria were; fulfilment of any other substance use disorder except AUD and nicotine use disorder; frequent use (more than once a week) of any illicit drug during six months prior to inclusion, indication of a significant somatic risk associated with alcohol consumption e.g. significantly elevated liver enzymes (AST, ALT, GGT), ongoing treatment for AUD, a major psychiatric condition e.g. severe major depression or non-treated bipolar disorder.

Participants were included at or in conjunction with, the screening occasion. All participants signed an informed consent form and were thereafter randomly allocated to either BSCT or MET. Participants were included at or in conjunction with, the screening occasion. All interested individuals signed an informed consent form and were thereafter randomly allocated to either BSCT or MET. Clinical data was collected during their visits to the study coordinator in the first 172 participants. Due to the pandemic, the study procedure had to be altered in March 2020. This meant that for the remaining 78 participants baseline-screening procedure was conducted via video. This implied that they used either a tablet or a smartphone to participate in the meeting. The follow-up interviews were also conducted via video, and self-report instruments (i.e. the clinical data) was thereafter collected by mail, and sent to the research coordinator via pre-paid envelopes.

### Interventions

#### Behavioral self-control training

The BSCT manual consisted of five sessions based on cognitive and behavior therapy, modified from the self-help manual by Miller and Muñoz [12, 71] into a Swedish clinicians’ manual [60]. BSCT themes were goal setting and motivation, identifying risk situations; skills training for moderation and increasing days with abstinence; maintenance of reduced consumption. Homework assignments in line with session content were included between sessions as part of the intervention.

#### Motivational enhancement therapy

The MET manual was originally developed as part of the project MATCH trial [15].In the current study, a version adapted to a Swedish addiction treatment context was used [13]. The MET manual contained four sessions where the first session included feedback on initial assessments, followed by three MI sessions. Two optional worksheets were included, the first aimed to support the formulation of a change plan, and the second maintenance of change.

Both interventions were finalized within a treatment period of 12 weeks. Treatments were initially carried out face-to-face. Due to the Covid pandemic, the study procedure was altered in March 2020, which meant that the participants received treatment through video-meetings from March 2020 until the finalization of patient recruitment in December 2020. In all, 78 patients received treatment via video.

The participants who requested additional treatment at the 26-week follow-up received either pharmacotherapy and/or additional psychological treatment after this follow-up. Additional treatment and the patients’ stated treatment goal (CD/abstinence) were registered at the follow-ups.

### Measures

#### Outcome measures

The following outcome measures concern both outcomes for the 52-week timepoint, and are included as variables in the predictor analyses.

#### Baseline characteristics

Sociodemographic data were collected and psychiatric diagnostics was assessed by the MINI-interview (DSM-5) (American Psychiatric Association, 2013). The MINI-interview included the assessment of AUD according to the DSM-5. Alcohol use disorder as an outcome, was also assessed by the MINI-interview at 52 weeks post inclusion.

#### Alcohol consumption

The following variables were investigated as outcomes. Change in mean weekly alcohol consumption between baseline and 52 weeks was measured by the Timeline Follow-Back method (TLFB) 30 days [61]. Alcohol consumption was measured by the number of standard drinks by Swedish standards (12 g of pure ethanol) consumed per day. The mean number of standard drinks per week during the 30-day period was calculated by dividing the total number of drinks by 4.29 weeks (number of weeks in 30 days). CD was defined as having a mean weekly alcohol consumption < 10.0 standard drinks per week (for both women and men). The definition of CD as in line with the consumption level of < 10.0 standard drinks was chosen as this level represent the newly decided low-risk drinking levels in Sweden. This cutoff does not differentiate between individuals who currently abstain from alcohol and those with any alcohol consumption. Heavy drinking days was defined as the percentage of occasions with a consumption of ≥ 4/ ≥ 5 standard drinks for women/men of the total 30 days measured. Carbohydrate-deficient transferrin (CDT) and phosphatidylethanol (PEth) were assessed as objective measures of alcohol consumption.

#### Clinical characteristics

The following self-report instruments were used to collect data on clinical characteristics. Self-rated degree of alcohol problems was assessed by the Alcohol Use Disorders Identification Test (AUDIT) [62]. Alcohol related harm was assessed by the Short Index of Problems (SIP) [63]. Family history of AUD was measured by a subscale from the Addiction Severity Index [64]. Impaired control over alcohol consumption was measured by two subscales in the Impaired Control Scale (ICS) (Failed Control, Perceived Control) [65, 66]. Alcohol craving was assessed with the Penn Alcohol Craving Scale (PACS) [67]. Depressive and anxiety symptoms were assessed with the Montgomery Asberg Depression Rating Scale-Self Reported (MADRS-S) [68] and the Generalized Anxiety Disorder Assessment (GAD-7) [69]. Difficulties in emotion regulation was measured with the Difficulties in Emotion Regulation Scale 16 items (DERS-16) [70]. Motivation for change was measured by the Visual Analogue Scales 1) Importance and; 2) Competence, with both scales ranging from 1 to 10 [71]. Further, use of nicotine products was assessed at baseline and included individuals who were regular users of tobacco (including snuff) and nicotine replacement products. Treatment satisfaction was measured post treatment (12 weeks), and 52 weeks by the Client Satisfaction Scale (CSQ-8) [72]. Lastly, data on if participant’s sessions were conducted face-to-face or as a video intervention was collected by the study-therapist.

### Outcomes in predictor analyses

Three variables were chosen as outcomes in the predictor analyses. These were: mean weekly alcohol consumption, proportion of participants drinking according to definition of CD (< 10.0 standard drinks per week for both genders) and number of AUD criteria according to the DSM-5, all three measures defined as change between baseline and 52 weeks.

### Statistical analyses

Statistical analyses for the 52-week outcomes and regression models were produced by a professional statistician outside the research team.

#### Power calculation

The power calculation of the current trial was originally conducted for the primary outcome and was presented in a previous publication [59].

#### Statistical programs and packages

Basic statistical computations were conducted with IBM SPSS Statistics for Windows, Version 26.0. Armonk, NY: IBM Corp, released 2019. Linear mixed effects models, and multiple imputation were estimated with the statistical programme R version 4.1.1 [73], package nlme brms [74] and mice [75]. Marginal means and effect sizes along with their confidence intervals were estimated with the R package ‘emmeans’ Emmeans: Estimated Marginal Means, aka Least-Squares Means. R package version 1.7.2. [76].

#### Analytical plan

Analyses of outcomes were based on an Intention to treat principle. The between group outcomes at 52 weeks were analyzed with linear and logistic regression models. For each continuous outcome a mixed linear regression was used, including the change from baseline to follow-up as the dependent variable. For each binary outcome, a mixed logistic regression was used, with the observed values as the dependent variable. For all models, included independent variables were the baseline value, treatment group, timepoint and treatment x timepoint interaction. A random intercept for every unique participant was included to account for the repeated measures in all models. The results from all models are presented with a 95% confidence interval and a p-value for the difference between groups in change from baseline to 52 weeks.

To handle missing data at the 52nd week follow-up, we conducted a multiple imputation for all included independent variables which had any missing data. This was done by pooling the results of analyses performed in five imputed datasets. The imputation was performed using baseline variables and variables from the same time point as the imputed variable. All mixed regression analyses were also performed on non-imputed data to be able to compare the results to imputed data. Further, adjustment for gender was included in models for both imputed and non-imputed data, as there was a gender imbalance between treatment groups (more women in MET) which potentially could affect the results.

For the predictor analyses, we used stepwise linear and logistic regression models. Univariable regression models were conducted for all chosen predictors (covariates), based on their baseline values. First, univariable predictors were identified. Variable selection was performed by first combining all five datasets. These were then included in a multivariable model, according to a forward selection model, meaning that variables were included in the model, one by one. The covariate that generated the best improvement of the model in each step, was selected. The selection was based on the Akaike Information Criteria (AIC) criteria [77]. The formula of AIC is; AIC = −2(log-likelihood) + 2 K, in which K stands for the number of variables included in the model, including the intercept. In this formula, the measure of log-likelihood is the measure of model fit. The AIC was thus used to compare the relative strength of different models, where a lower number means a better fit. Further, non-significant variables could also be included in the multivariable model, if it improved the AIC value. This meant that we accepted predictors in the multivariable models without statistical significance below 0.05, if they improved the goodness of fit. The predictor analyses were based on multiple imputed data as described previously.

## Results

### Baseline characteristics

Baseline sociodemographic and clinical characteristics for the sample is presented in Table 1. In this sample, 48% were women, the mean age was approximately 52 years and mean number of AUD criteria fulfilled was 5.4, indicating a moderate alcohol use disorder. The level of psychiatric comorbidity was low regarding both depressive and anxiety symptoms, and well below clinical cutoffs. A total of 19.5% of the participants in BSCT and 29.5% in MET reported that they had received additional treatment (pharmacological and/or psychological) (t = 3.462, *p* = 0.177) after the 26-week follow-up (not displayed in table).

**Table 1:** Baseline descriptive and clinical characteristics between groups (observed values)

	BSCT (n = 125)		MET (n = 125)	
Gender (percentage female)	42.0		54.0	
	Mean	SD	Mean	SD
Age (years)	52.2	11.4	51.4	10.7
Alcohol use disorder (DSM-5 criteria fulfilment)	5.39	1.93	5.14	2.03
	n	%	n	%
Alcohol use disorder (DSM-5)				
Mild	23	18.4	31	24.8
Moderate	43	34.4	41	32.8
Severe	59	47.2	53	42.4
Marital status				
Married/Partner	91	73.9	92	74.1
Divorced/Widowed	9	7.3	10	8.0
Single	23	18.9	22	17.7
Educational status				
Basic education	5	4.1	3	2.6
Upper secondary school	24	19.5	17	13.8
Post-secondary education	12	9.8	14	11.4
University < 3 years	20	16.3	27	22.0
University > 3 years	62	50.4	62	50.4
Occupational status				
Employed/self-employed	98	79.7	106	86.2
Retired/housekeeper	18	14.6	9	7.3
Student/parental leave	1	0.8	2	1.6
Unemployed/sick leave	6	4.9	6	4.9
	Mean	SD	Mean	SD
Mean weekly consumption (standard drinks) (TLFB 30 days)	22.27	12.91	23.03	13.31
Mean number of heavy drinking days (of total 30 days)	10.13	7.69	11.71	9.02
	n	%	n	%
Percentage of individuals at a controlled drinking level (< 10 weekly standard drinks)	12	9.6	14	11.2
Percentage of individuals with former AUD treatment > 3 months	26	20.8	33	26.4
Percentage of individuals with ongoing antidepressant medication ≥ 30 days	30	24.0	33	26.0
Percentage of individuals with a family history of AUD (missing values = 9)	76	66.1	74	64.3
Nicotine use (cigarettes, snuff, nicotine replacement)	43	34.4	50	40.0
	Mean	SD	Mean	SD
PEth	0.58	0.51	0.60	0.52
CDT	2.05	1.24	2.14	1.58
AUDIT	18.86	5.74	19.15	5.87
SIP	11.80	6.09	11.91	6.85
ICS (FC + PC)	35.69	10.42	34.92	9.93
PACS	8.27	5.52	8.35	5.58
MADRS-S	9.00	6.86	9.65	7.03
GAD 7	3.28	4.01	3.43	3.61
DERS-16	29.11	11.15	28,17	10,22
Motivation to change VAS (1–10)				
a) Importance	9.22	1.06	9.33	0.92
b) Self-efficacy	7.42	1.76	6.99	2.11
EQ-5D 3L	5.91	0.91	6.01	1.06
EQ-5D VAS (1–100)	72.84	13.61	72.72	12.39
Sessions attended before 26 weeks	4.40	1.44	4.06	1.63

*DSM-5* Diagnostic and Statistical Manual of Psychiatric disorders, 5th Edition, *TLFB* Timeline Followback, *AUD* Alcohol use disorder, *PEth* phosphatidylethanol, *CDT* carbohydrate-deficient transferrin, *AUDIT* Alcohol Use Disorders Identification Test, *SIP* Short Index of Problems, *ICS* Impaired Control Scale, *FC* Failed Control), *PC* Perceived Control, *PACS* Penn Alcohol Craving Scale, *MADRS-S* Montgomery Asberg Depression Rating Scale-Self Rated, *GAD 7* Generalized Anxiety Disorder Assessment, *DERS 16* Difficulty of Emotion Regulation-Short Scale 16 items, *Motivation to change VAS* Motivation to change, Visual Analogue Scale, *EQ5D-3L* European Quality of Life 5 Dimensions 3 Level Version, *EQ 5D VAS* European Quality of Life 5 Dimensions 3 Level Version, Visual Analogue Scale

At the 52-week follow-up, a total of 72.8% (n = 182) of the participants attended the follow-up meeting, with no difference in follow up rate between treatment groups (BSCT n = 87, MET n = 95) (t = 1.29, *p* = 0.320). Out of these, 74% (n = 135) left blood samples for the evaluation of alcohol biomarkers (PEth, CDT). A comparison between non-imputed and multiple imputed results for the outcome of CD is presented in Table 2. The results showed that the estimated model-based mean values showed corresponding results between the two different analytical methods. Hence, the results of alcohol consumption outcomes, such as the proportion of CD may be assumed to be robust against missing data.

**Table 2:** Multiple regression models with 95% confidence intervals for the outcome proportion of individuals with CD across behavioral self-control training and motivational enhancement therapy at the 52-week follow-up

Outcome	n	BSCT	MET
		Estimate ( 95% CI)	Estimate ( 95% CI)
Proportion of individuals with CD (MI, A)	250	0.57 (0.47–0.67)	0.44 (0.35–0.52)
Proportion of individuals with CD (NI, A)	182	0.54 (0.48–0.62)	0.45 (0.36–0.51)
Proportion of individuals with CD (NI, NA)	182	0.52 (0.47–0.59)	0.46 (0.38–0.51)

*MI,A* Multiple imputation, adjusted for gender, *NIA* Non-imputed data, adjusted for gender, *NI,NA* Non-imputed data, not adjusted for gender

### Outcomes between BSCT and MET at 52 weeks

There was a statistically significant difference between groups regarding the change in percentage of participants drinking according to definition of CD from baseline to 52 weeks favoring BSCT, when adjusting for gender (Table 3). Treatment satisfaction was also higher in BSCT compared to MET at 52 weeks.

**Table 3:** Outcomes at baseline and 52 weeks post inclusion across BSCT and MET

	Baseline				52 weeks				Change between groups from baseline and 52 weeks		*P*
	BSCT n = 125		MET n = 125		BSCT		MET		BSCT	MET	
	Estimate	95%-CI	Estimate	95%-CI	Estimate	95%CI	Estimate	95% CI	Estimate (95% CI)	Estimate (95% CI)	
Mean weekly alcohol consumption 30 days	21.97	20.16; 23.79	23.13	21.34;24.92	12.53	10.42;14.65	13.86	11.88–15.84	−9.57 (−12.6;6.99)	−9.23 (−11.66;−6.79)	0.839
Percentage of heavy drinking days (of total 30 days)	33.71	29.72; 37.70	38.99	35.01;42.96	18.96	13.85;24.06	18.41	14.15; 22.68	−15.33 (−21.36 to −9.31)	−20.36 (−25.68 to −15.04)	0.207
Proportion with controlled drinking (< 10.0/ < 10.0)	0.09	0.05; 0.17	0.10	0.05; 0.17	0.57	(0.47; 0.67)	0.44	0.35; 0.52	0.47 (0.36 to 0.57)	0.34 (0.25 to 0.43)	0.048*
AUD criteria DSM-5	5.36	4.99; 5.73	5.14	4.78; 5.51	2.46	1.99; 2.93	2.94	2.49; 3.40	2.47 (1.98 to 2.96)	2.94 (2.47 to 3.41)	0.126
PEth	0.63	0.53; 0.74	0.64	0.54; 0.73	0.48	0.32; 0.63	0.49	0.34; 0.63	−0.17 (−0.34 to 0.01)	−0.15 (−0.31 to 0.01)	0.825
CDT	2.11	1.88; 2.35	2.10	1.86; 2.34	1.99	1.67; 2.31	2.15	1.76; 2.55	−0.12 (−0.47 to 0.23)	0.05 (−0.31 to 0.42)	0.368
AUDIT	18.87	17.86; 19.88	19.13	18.10; 20.16	12.54	10.57; 14.51	13.00	11.04; 14.96	−6.32 (−8.32; −4.32)	−6.13 (−7.96; −4.31)	0.820
SIP	11.71	10.63; 12.80	11.92	10.82; 13.03	6.08	4.81; 7.35	6.51	5.40; 7.62	−5.57 (−6.96; −4.18)	−5.44 (−6.73; −4.14)	0.886
PACS	8.34	7.35; 9.32	8.25	7.28; 9.22	5.69	4.25; 7.12	4.92	3.39; 6.45	−2.76 (−4.32; −1.19)	−3.29 (−4.80: −1.78)	0.606
ICS (FC + PC)	35.56	33.52; 37.59	34.83	32.80; 36.86	24.05	20.48; 27.62	24.40	21.91; 26.89	−11.65 (−15.20 to −8.10)	−10.38 (−12.98 to −7.78)	0.563
MADRS-S	9.05	7.82; 10.27	9.62	8.40; 10.84	8.20	5.59; 10.81	8.49	6.34; 10.64	−0.92 (−3.61 to 1.77)	−1.10 (−3.24 to 1.03)	0.876
GAD7	3.39	2.64; 4.15	3.43	2.69; 4.18	3.78	2.48; 5.08	3.90	2.54; 5.25	0.33 (−0.95 to 1.61)	0.48 (−0.93 to 1.90)	0.863
EQ 5D-3L	5.93	5.76; 6.11	6.01	5.84; 6.19	5.95	5.70; 6.20	5.91	5.67; 6.16	0.01 (−0.26 to 0.28)	−0.10 (−0.36 to 0.17)	0.569
EQ 5D VAS	72.65	70.17; 75.13	72.79	70.33; 75.25	74.46	70; .4178.50	77.43	73.63; 81.22	1.81 (−2.20; 5.82)	4.63 (0.73; 8.54)	0.198
Motivation for change											
Importance	9.23	8.90; 9.56	9.31	8.98; 9.65	7.60	6.92; 8.28	7.97	7.41; 8.53	−1.63 (−2.32; −0.95)	−1.34 (−1.90; −0.78)	0.426
Competence	7.41	7.06; 7.76	6.97	6.61; 7.32	7.72	7.23; 8.20	7.59	7.19; 7.99	0.29 (−0.25; 0.84)	0.63 (0.20; 1.06)	0.299

	12 weeks				52 weeks				Change between baseline and 52 weeks		*P*
	BSCT		MET		BSCT		MET		BSCT	MET	
CSQ 12 weeks	26.31	25.42; 27.21	24.17	23.17; 25.16	25.27	23.93; 26.60	23.73	22.55; 24.92	25.77 (24.87; 26.67)	23.61 (22.73; 24.49)	< 0.001***

The outcome of controlled drinking (< 10/ < 10) weekly standard drinks (women/men) was calculated as a dichotomous variable and is displayed in a proportion of individuals who attained this drinking level at 52 weeks

Client Satisfaction measured (Client Satisfaction Questionnaire) at 12 and 52 weeks across BSCT and MET

*AUD* Alcohol use Disorder, DSM-5 = *CDT* carbohydrate-deficient transferrin, *PEth* phosphatidylethanol, *GGT* gamma-glutamyl transferase, *AST* aspartate amino transferase, *ALT* alanine amino transferase, *AUDIT* Alcohol Use Disorders Identification Test, *SIP* Short Index of Problems, *ICS* Impaired Control Scale, *PACS* Penn Alcohol Craving Scale, *MADRS-S* Montgomery Asberg Depression Rating Scale-Self Rated, *GAD 7* Generalized Anxiety Disorder Assessment, *Motivation to change VAS* Motivation to change, Visual Analogue Scale, *EQ 5D VAS* EQ 5D Visual Analogue Scale

^*^p < 0.05, **p ≤ 0.01, ***p < 0.001

### Predictors of treatment outcome

In Tables 4, 5, 6, the results of the predictor analyses are presented.

**Table 4:** Univariable and multivariable predictors of mean weekly alcohol consumption at the 52 week follow-up

	Univariable				Multivariable			
	Estimate	95% CI	*P*	R Square	Estimate	95%-CI	*P*	R Square
								0.12
Proportion of heavy drinking days	−0.02	(−0.13 to 0.09)	0.733	0.07				
Alcohol use disorder (DSM -5)	−0.08	(−0.74 to 0.58)	0.814	0.07				
AUDIT	0.12	(−0.09 to 0.33)	0.270	0.07				
Impaired control (ICS) (FC + PC)	0.03	(−0.11 to 0.16)	0.694	0.07				
Motivation to change *Importance*	−0.73	(−2.22 to 0.76)	0.323	0.07				
Motivation to change *Competence*	−0.31	(−1.33 to 0.71)	0.517	0.07				
Alcohol craving	0.00	(−0.31 to 0.32)	0.986	0.07				
Difficulty of emotion regulation (DERS-16)	−0.00	(−0.14 to 0.14)	0.994	0.07				
Depressive symptoms (MADRS-S	−0.00	(−0.25 to 0.25)	0.979	0.07				
Sessions attended	−0.81	(−1.87 to 0.24)	0.123	0.09	−0.90	(−1.95 to 0.15)	0.087	
Family history of AUD	−0.15	(−3.13 to 2.83)	0.919	0.07				
Nicotine user	1.61	(−1.55 to 4.77)	0.305	0.08				
Income	−0.00	(−0.00 to 0.00)	0.945	0.07				
Previous treatment	2.32	(−0.57 to 5.20)	0.115	0.08				
Age	−0.02	(−0.19 to 0.15)	0.833	0.07				
Gender (female)	−3.34	(−7.40 to 0.73)	0.097	0.10	−3.57	(−7.55 to 0.41)	0.074	
Condition (MET)	0.79	(−1.66 to 3.23)	0.526	0.07				
Video intervention	−0.86	(−4.62 to 2.91)	0.635	0.07				
Baseline mean weekly consumption (TLFB 30 days)	0.20	(0.07–0.33)	0.005**	0.07	0.18	(0.05–0.32)	0.012*	

*DSM 5* Diagnostic and Statistical Manual of Mental Disorders, 5th edition, *AUDIT* Alcohol Use Disorders Identification, *ICS* Impaired Control Scale, *FC* Failed Control, *PC* Perceived Control Test, *VAS* Visual Analogue Scale, *PACS* Penn Alcohol Craving Scale, *DERS 16* Difficulty of Emotion Regulation-Short Scale 16 items, *MADRS-S* Montgomery Asberg Depression Rating Scale-Self Rated, *AUD* Alcohol use disorder, *MET* Motivational Enhancement Therapy, *TLFB* Timeline FollowBack 30 days

^*^ ≤ 0.05 ** ≤ 0.01

**Table 5:** Univariable and multivariable predictors of controlled drinking (< 10.0 standard drinks) at the 52 week follow-up

	Univariable analysis			Multivariable analysis		
	Odds ratio	95% CI	*P*	OR	95% CI	*P*
Alcohol use disorder (DSM-5)	1.01	(0.83–1.22)	0.934			
AUDIT	0.98	(0.91–1.07)	0.675			
Impaired control (ICS) (FC + PC)	1.00	(0.95–1.05)	0.929			
Motivation to change importance (VAS 1–10)	1.04	(0.74–1.48)	0.806			
Motivation to change competence (VAS 1–10)	1.09	(0.90–1.32)	0.346			
Alcohol craving (PACS)	1.00	(0.93–1.07)	0.955			
Difficulty of emotion regulation (DERS-16)	1.00	(0.97–1.04)	0.843			
Depressive symptoms (MADRS-S)	1.00	(0.94–1.05)	0.859			
Sessions attended	1.02	(0.86–1.21)	0.811			
Family history of AUD	0.76	(0.44–1.31)	0.315			
Nicotine user	0.74	(0.41–1.32)	0.302			
Income	1.00	(1.00–1.00)	0.834			
Previous treatment	0.74	(0.38–1.42)	0.357			
Age	1.00	(0.96–1.03)	0.744			
Gender	1.65	(0.83–3.30)	0.145	1.80	(0.87–3.73)	0.106
Condition (MET)	0.59	(0.32–1.08)	0.085	0.54	(0.29–1.03)	0.059
Video intervention	1.52	(0.85–2.74)	0.158			
Baseline controlled drinking (TLFB 30 days)	2.97	(0.91–9.73)	0.071	3.13	(0.95–10.39)	0.061

*DSM 5* Diagnostic and Statistical Manual of Mental Disorders, 5th edition, *AUDIT* Alcohol Use Disorders Identification, *ICS* Impaired Control Scale, *FC* Failed Control, *PC* Perceived Control Test, *VAS* Visual Analogue Scale, *PACS* Penn Alcohol Craving Scale, *DERS 16* Difficulty of Emotion Regulation-Short Scale 16 items, *MADRS-S* Montgomery Asberg Depression Rating Scale-Self Rated, *AUD* Alcohol use disorder, *MET* Motivational Enhancement Therapy, *TLFB* Timeline FollowBack 30 days

**Table 6:** Univariable and multivariable predictors of recovery from alcohol use disorder at the 52 week follow-up. week post inclusion

	Univariable analysis				Multivariable analysis			
Independent variables	Estimate	95% CI	*P*	R square	Estimate	95% CI	*P*	R square
								0.14
Proportion of heavy drinking days	0.00	(−0.01 to 0.02)	0.653	0.09				
Baseline Alcohol use disorder (DSM 5)	0.34	(0.14–0.54)	0.002**	0.09	0.25	(0.04–0.47)	0.024*	
AUDIT	0.03	(−0.11 to 0.17)	0.618	0.10				
Impaired control (FC + PC)	0.04	(0.01–0.08)	0.013*	0.12	0.04	(0.01–0.08)	0.012*	
Motivation for change importance (VAS 1–10)	−0.03	(−0.40 to 0.35)	0.883	0.09				
Motivation for change competence (VAS 1–10)	−0.12	(−0.26 to 0.01)	0.076	0.10				
Alcohol craving (PACS)	0.05	(−0.01 to 0.11)	0.090	0.11				
Difficulty of emotion regulation (DERS-16)	−0.01	(−0.04 to 0.01)	0.374	0.09				
Depressive symptoms (MADRS-S)	0.01	(−0.05 to 0.06)	0.760	0.09				
Sessions attended	0.06	(−0.28 to 0.39)	0.709	0.10				
Family history of AUD	−0.10	(−0.69 to 0.49)	0.744	0.09				
Nicotine user	0.10	(−0.51 to 0.71)	0.747	0.09				
Income	0.00	(−0.00 to 0.00)	0.342	0.09				
Previous treatment	0.65	(−0.16 to 1.47)	0.111	0.11				
Age	0.02	(−0.02 to 0.06)	0.291	0.10				
Gender	-0.03	(−0.60 to 0.54)	0.917	0.09				
Condition (MET)	0.53	(−0.06 to 1.13)	0.079	0.11	0.54	(−0.05 to 1.13)	0.073	
Video intervention	0.01	(−0.95 to 0.96)	0.987	0.09				
Baseline mean weekly consumption (TLFB 30 days)	0.01	(−0.02 to 0.03)	0.659	0.09				

*DSM 5* Diagnostic and Statistical Manual of Mental Disorders, 5th edition, *AUDIT* Alcohol Use Disorders Identification, *ICS* Impaired Control Scale, *FC* Failed Control, *PC* Perceived Control Test, *VAS* Visual Analogue Scale, *PACS* Penn Alcohol Craving Scale, *DERS 16* Difficulty of Emotion Regulation-Short Scale 16 items, *MADRS-S* Montgomery Asberg Depression Rating Scale-Self Rated, *AUD* Alcohol use disorder, *MET* Motivational Enhancement Therapy, *TLFB* Timeline FollowBack 30 days

^*^ =  < 0.05 ** =  < 0.01

#### Mean weekly alcohol consumption

For the outcome mean weekly alcohol consumption at 52 weeks, the univariable analysis identified one statistically significant predictor, where lower baseline weekly consumption predicted a lower mean weekly consumption at 52 weeks (Table 4). In the multivariable model, weekly consumption at baseline was maintained as predictor while female gender (p = 0.074) and number of treatment sessions attended (p = 0.087) were added to the model. This meant that more sessions attended and female gender improved the goodness of fit of the model but were not statistically significant in the multivariable analysis. Together, the predictors explained 12% of the variance of the model. Only weekly alcohol consumption was statistically significant in the multivariable analysis.

#### Controlled drinking

The univariable analyses for the outcome of CD at 52 weeks revealed no statistically significant predictors (Table 5). In the multivariable model, CD at baseline (p = 0.061) together with female gender p = 0.106) and having BSCT as treatment (p = 0.059) were improving the goodness of fit, a but none were reaching the 0.05 significance level.

#### Alcohol use disorder

For the prediction of reduction in AUD symptoms at 52 weeks, no variables were significant predictors in the univariable analyses (Table 6). In the multivariable model, a lower level of AUD severity (number DSM-5 criteria) at baseline (p = 0.024) predicted, and a lower level of impaired control over alcohol consumption predicted reduction in symptoms of AUD. Further, receiving BSCT was found to improve the model but was not statistically significant (p = 0.073). The three variables collectively explained 14% of the variance of the model.

## Discussion

The aims of the present study were to investigate differences in one-year outcomes between BSCT and MET, and to identify predictors of favorable treatment outcome in individuals with AUD, with a treatment goal of CD. The results showed that BSCT was superior to MET regarding the change in percentage of individuals who attained CD at 52 weeks. Participants in BSCT also reported higher treatment satisfaction (CSQ 8) at 52 weeks. A lower baseline consumption level predicted lower consumption at 52 weeks, -and a less severe AUD at baseline predicted a lower level of AUD symptoms at 52 weeks. Female gender, attending more treatment sessions and receiving BSCT as opposed to MET, contributed to the models* reduction in weekly alcohol consumption and attaining CD at 52 weeks. Moreover, a lower level of impaired control over alcohol consumption, and a lower baseline level of AUD predicted a reduction of symptom burden of AUD at 52 weeks.

As hypothesized, the current study corroborated previous research in showing that a lower pretreatment consumption at baseline predicted a favorable treatment outcome [29, 32, 56, 57]. These findings have important clinical implications. First, the results point to the importance of close monitoring of consumption levels during treatment and the provision of support to patients in reducing consumption early in the treatment phase. Support may e.g., be provided by guiding patients’ attention to a specific consumption goal e.g., drinks per drinking day and weekly consumption levels, as suggested in the initial session of BSCT [11, 60].

In previously presented results for the primary outcome of mean weekly consumption at 26 weeks in the current trial, there were no identified differences between groups [59]. The only detected difference between conditions were the proportion with weeks with hazardous drinking, favoring BSCT. In the current study we wanted to explore if there were differences on long-term outcomes between the two treatments. In line with our superiority hypothesis, BSCT was superior to MET in attaining CD at 52 weeks, and treatment satisfaction. The study applied the definition of CD as a stable pattern of drinking in line with low-risk drinking, and therefore used the current low-risk drinking levels in Sweden as a cutoff for CD. This also included individuals with no alcohol consumption the past 30 days (n = 11 from a total of 182) as CD may involve periods of abstaining from alcohol, although the stated goal is CD.

One plausible explanation to the superior results for BSCT is that BSCT emphasizes low-risk drinking levels as part of goal-setting, together with moderation strategies to obtain a goal of CD [11, 12]. Another explanation to why a higher percentage of BSCT participants obtained a CD goal compared to MET, may be that in the BSCT manual, patients are encouraged to state a specific consumption goal. Formulating a specific consumption goal, e.g., a maximum number of drinks per day and/or week has been proposed to result in outcomes closer to CD as compared to when patients state less specific treatment goals [7, 38]. Moreover, another difference in methodology between BSCT and MET is that BSCT includes moderation skills training. The MET manual does not include any specific guidance on how to reach goals to reduce drinking or how to abstain from alcohol, which may be less favorable when aiming for a CD goal as opposed to abstinence. In a qualitative interview study from the current trial on patients’ experiences from receiving MET, the results indicated that some of the patients found the treatment to lack specific guidance on how to attain a CD goal [78]. These results taken together indicate that the MET manual may need modification to also suit both abstinent- and CD treatment goals.

Female gender was not statistically significant as a predictor for the reduction of weekly consumption or CD at 52 weeks although it contributed with substantial effect in both models. Still, data showed that women were 80% more likely to attain a goal of CD than men at 52 weeks. Previous studies have shown conflicting results regarding gender differences in AUD treatment [45–48]. One factor that varied between genders in previous studies is level of AUD severity [21, 23]. In the current study, women had a lower level of AUD (5.0 criteria, SD = 1.9), at baseline compared to men (5.5, SD = 2.0), (t = 2.120,* p* = 0.035), but severity of AUD did not fit into the multivariable model for CD or mean weekly consumption at 52 weeks. These results indicate that the comparably lower severity of AUD in women at baseline was not contributing to the favorable outcome in this study. Another factor that could potentially have contributed to gender differences, was baseline consumption levels, since differences in consumption level at start of treatment is predictive of treatment outcome [29, 32]. In our study, baseline consumption was included in the model, thus adjusted for, which suggests that women were more successful than men, irrespective of baseline consumption levels. Further strengthening the conclusion that gender predicted treatment outcome was that other clinically relevant factors did not fit the model either, e.g. motivation for change, age, craving, treatment attendance, or family history of AUD. Moreover, there were no other differences between genders with regards to clinical and baseline characteristics (not displayed in table). In summary, we cannot provide any plausible explanation to gender differences in our results, which warrants for more research on gender specific differences and its possible role on how CD is attained.

In the current study, a higher number of sessions attended was included in the prediction model for mean weekly consumption at 52 weeks, adjusting for treatment condition and baseline alcohol consumption. Patients who were completers, i.e. retained in the planned treatment hence had more favorable outcomes, irrespective of treatment received or baseline consumptions patterns [44]. The result may also implicate that those who are successful in reducing their consumption are more prone to retain in treatment. Regular treatment evaluation, or a more systematic use of feedback-informed treatment methods may increase the possibility for therapists to address patients’ dissatisfaction or doubts of the treatment’s effectiveness [79].

The study identified that a lower baseline AUD severity and a lower level of impaired control predicted a reduction in AUD (DSM-5) symptom burden at 52 weeks. Self-rated impaired control, both in regard to limiting alcohol consumption at specific occasions, and abstaining, has been predictive of outcome both in population-based samples and in moderation-oriented AUD treatment in a clinical sample [41, 80–82]. Impaired control as a specific feature of AUD, has been suggested to be under-addressed in AUD research and treatment [41, 65, 83, 84]. Our results support IC as a relevant feature to assess before treatment and to continuously evaluate as a measure of treatment effect in CD-oriented treatments.

The absence of dependence severity as a predictor of drinking outcomes was a surprising negative finding in the present study, since several previous studies have shown an association between dependence severity and alcohol consumption [50, 85, 86]. For example, in a Swedish population based study, both women and men with the highest symptom burden (≥ 5 dependence criteria) (DSM IV) were largely represented in the highest consumption category (> 18/ > 28 weekly standard drinks) [86]. Despite this association, the same study showed that a proportion of severely dependent (54% of women and 42% of men) were represented in the low-to moderate consumption category. This study illustrates that the clinical presentations vary substantially within treatment-seeking individuals with AUD with regards to consumption levels, and ability to limit and control their drinking. Instead, baseline level of AUD may be a stronger predictor of reduction in AUD severity in a treatment setting, as shown in the present study. Opposed to our hypothesis, craving was not identified as a predictor of outcome in the current study. Craving is associated with relapse in abstinence outcomes, e.g. after residential treatment, but is less supported in outcomes for CD [21, 87–89]. Craving may thus be influenced by the severity of the disorder, and hence be less prominent in the current sample as compared to clinical samples in inpatient- or residential treatment.

Lastly, we found it noteworthy that participating in treatment via video or face-to-face did not predict treatment outcome, corroborating the results for the primary endpoint of 26 weeks from the current trial [59]. Taken together, these results demonstrate that video-based interventions may be an option to patients with AUD who may hesitate to seek regular face-to-face treatment or their access to care is restricted by e.g., long distances, work or any disability.

### Strengths and limitations

The current study has strengths and limitations worth mentioning. To our knowledge, this is the first RCT investigating the efficacy of BSCT and MET in patients with AUD with the aim of CD. It included a fairly large clinical representative sample with a balanced gender distribution. As the original power calculation was not conducted with this study objective in mind, the multiple testing in the current study is a limitation. One reason for including a broad range of variables was to reduce the risk of excluding a potential predictor which would be important to outcomes. This means that the detected results need to be interpreted cautiously, and need further corroboration in future studies. Another limitation is that the chosen selection criteria in the RCT reduced the possibility to investigate some factors which may potentially have been clinically relevant to the investigated outcomes. For example, comorbid substance use disorders and severe psychiatric comorbidity such as nontreated bipolar disorder, and inpatient treatment for detoxification were listed as exclusion criteria. Hence, the results from this study, may only be inferred to treatment seeking individuals with AUD with a low degree of psychiatric comorbidity. Given the heterogeneity of AUD we want to stress the importance of *not* inferring the study results to the total population of AUD. However, we propose that one major strength of the current study in being an RCT means that the results are highly transferrable to the corresponding subpopulation in AUD. Lastly, the BSCT and MET manuals contained an unequal number of sessions (five versus four), which implies that treatment intensity differed between the conditions. This difference was deemed to be acceptable, as MET in previous studies has shown to be equally efficacious compared to more extensive treatments. However, the actual number of treatment sessions delivered was not different between groups, as an extra session was more commonly added in the MET condition, compared to in the BSCT arm. The aforementioned qualitative study on patient experiences on receiving MET as their treatment demonstrated that the treatment was perceived as too brief [78]. This may have affected the therapists’ decision to add an extra session to comparably more patients in the MET group, than what was the case in BSCT.

### Conclusions

The current study corroborated previous research on predictors of favorable treatment outcomes in AUD; being a lower baseline alcohol consumption and a lower level of impaired control over alcohol consumption, in treatment-seeking sample of AUD with low levels of psychiatric comorbidity. Moreover, the results showed that women were more likely to achieve a CD goal. New findings emerging from this study were that BSCT was superior compared to MET in supporting patients with AUD and a goal of CD to reach low-risk drinking, and to reduce symptom burden of AUD. Taken together, these clinical and demographic characteristics are of importance to take into consideration when both planning and evaluating treatment for CD in individuals with AUD. Future studies are warranted to further investigate what factors related to gender contribute to the observed differences.

## Data Availability

The datasets used and analyzed during the current study are available from the corresponding author on reasonable request.

## Abbreviations

AUD: Alcohol use disorder
CD: Controlled drinking
BSCT: Behavioral self-control training
COMBINE: Combined pharmacotherapies and behavioral interventions for alcohol dependence
UKATT: United Kingdom Alcohol Treatment Trial
AST: Aspartate aminotransferase
ALT: Alanine aminotransferase
GGT: Gamma-glutamyl transferase
CDT: Carbohydrate-deficient transferrin
PEth: Phosphatidylethanol
DSM-5: Diagnostic and Statistical Manual of Psychiatric Disorder, 5th edition
TLFB: Timeline follow-back
AUDIT: Alcohol use disorders identification test
SIP: Short Index of Problems
MADRS-S: Montgomery Asberg depression rating scale-self rated
GAD-7: Generalized Anxiety Disorder Assessment
PAC: Penn Alcohol Craving Scale
ICS: Impaired Control Scale
ASI: Addiction Severity Index
DERS-16: Difficulty of emotion regulation short scale 16 items
EQ 5D-3L: European quality of life 5 dimensions 3 level version
CSQ-8: Client satisfaction questionnaire 8 items
AIC: Akaike information criteria
